# Prognostic markers in cancer: the evolution of evidence from single studies to meta-analysis, and beyond

**DOI:** 10.1038/sj.bjc.6604999

**Published:** 2009-04-14

**Authors:** R D Riley, W Sauerbrei, D G Altman

**Affiliations:** 1 Department of Public Health, Epidemiology and Biostatistics, Public Health Building, University of Birmingham, Edgbaston, Birmingham B15 2TT, UK; 2 Institute of Medical Biometry and Medical Informatics, University Medical Center Freiburg, Stefan-Meier-Strasse 26, Freiburg 79104, Germany; 3 Centre for Statistics in Medicine, University of Oxford, Wolfson College Annexe, Linton Road, Oxford OX2 6UD UK

**Keywords:** prognosis, prognostic tumour marker, biomarker, primary studies, evidence synthesis, guidelines

## Abstract

In oncology, prognostic markers are clinical measures used to help elicit an individual patient's risk of a future outcome, such as recurrence of disease after primary treatment. They thus facilitate individual treatment choice and aid in patient counselling. Evidence-based results regarding prognostic markers are therefore very important to both clinicians and their patients. However, there is increasing awareness that prognostic marker studies have been neglected in the drive to improve medical research. Large protocol-driven, prospective studies are the ideal, with appropriate statistical analysis and clear, unbiased reporting of the methods used and the results obtained. Unfortunately, published prognostic studies rarely meet such standards, and systematic reviews and meta-analyses are often only able to draw attention to the paucity of good-quality evidence. We discuss how better-quality prognostic marker evidence can evolve over time from initial exploratory studies, to large protocol-driven primary studies, and then to meta-analysis or even beyond, to large prospectively planned pooled analyses and to the initiation of tumour banks. We highlight articles that facilitate each stage of this process, and that promote current guidelines aimed at improving the design, analysis, and reporting of prognostic marker research. We also outline why collaborative, multi-centre, and multi-disciplinary teams should be an essential part of future studies.

In oncology, prognostic markers (also called prognostic factors) are clinical measures used to help elicit an individual patient's risk of a future outcome, such as recurrence of disease after primary treatment. They play a key role in clinical practice, distinguishing patients into different risk groups and thus informing treatment strategies and aiding patient counselling. They can also be used to define strata in clinical trials to ensure comparability of treatment groups. Markers can be simple measures, such as the stage of disease or tumour size, but are often more complex, such as abnormal levels of proteins or genetic mutations. For example, within paediatric oncology, amplification of the MYCN proto-oncogene is a known indicator of poor outcome in neuroblastoma patients (Riley *et al*, 2004a). In adult oncology, it is well known that the number of positive lymph nodes has a strong influence on the prognosis of recurrence-free survival time in patients with primary breast cancer (Galea *et al*, 1992). For (nearly) all diseases, some important prognostic markers have been well established for a long time, but many more are investigated each year. Few, however, find a role in clinical practice.

Not all potential markers turn out to have prognostic value. Much research effort goes into conducting studies that evaluate the extent to which certain markers help clinical prognosis. Indeed, hundreds of prognostic marker studies are published in cancer journals each year. Unfortunately, though, there is large concern about the quality of such studies and it is clear that large progress is needed to produce clinically relevant results (Hinestrosa *et al*, 2007). Over more than 10 years, an increasing body of evidence has signalled that prognostic marker studies are often badly designed (Simon and Altman, 1994; Altman and Lyman, 1998), inappropriately analysed (Holländer and Sauerbrei, 2006), poorly reported (Riley *et al*, 2003; Kyzas *et al*, 2005), and subject to numerous biases, such as selective reporting (Kyzas *et al*, 2005, 2007a) and ‘optimal’ choice of cutpoints (Altman *et al*, 1994). Bad quality of design, analysis, and reporting of individual studies result in confusion regarding the prognostic value of a new marker (Sauerbrei, 2005). Furthermore, evidence-based marker results are a rarity, with systematic reviews and meta-analyses of multiple studies serving only to highlight the serious deficiencies within primary prognostic research (Altman, 2001). For example, two systematic reviews, one in neuroblastoma (Riley *et al*, 2004a) and another in non-small-cell-lung cancer (Brundage *et al*, 2002), found that over 100 different prognostic markers have been investigated in each field, yet the median number of articles per marker was only 1. Clinicians are thus faced with a confusing yet ever-growing body of literature, where new markers are regularly investigated but rarely in relation to existing markers. The evidence-base is further blurred by large heterogeneity across studies in the patient population, the method of measuring each marker, the outcomes reported, and the statistical analyses used, among numerous other factors (Riley *et al*, 2003), all of which limit a coherent meta-analysis.

In this paper, we examine the key methodological issues of the prognostic marker research process, encouraging a move towards higher standards and clinically useful results. Our aim is to discuss how high-quality evidence can evolve over time from initial exploratory studies, to large protocol-driven primary studies, to meta-analysis and even beyond, to large prospectively planned pooled analyses, and to the initiation of tissue banks. The work extends earlier articles aimed at a more statistical audience (Altman *et al*, 2006; Holländer and Sauerbrei, 2006; Sauerbrei *et al*, 2006; Riley *et al*, 2006a), and focuses on prognostic, rather than predictive, markers. In cancer, a prognostic marker is one that predicts a patient's clinical course, whereas a predictive marker is one associated with differential responses to a specific treatment (although this terminological distinction is perhaps not widespread across medicine). Predictive marker studies are relatively uncommon, and best embedded within randomised controlled trials. Sargent *et al* (2005) discuss how to conduct a clinical trial that assesses the utility of a predictive marker.

Our paper focuses on studies (or meta-analyses) that seek to evaluate a small number of pre-specified markers. In recent years, clinicians have become increasingly enthusiastic about the possibility of improving prognosis and prediction by applying the new technologies of measuring many thousands of genes. However, these technologies create a very large number of potential predictors and require statistical methods to extract a relatively small number of relevant items. We also do not consider the study of prognostic markers from high-dimensional data, such as microarray studies (Tinker *et al*, 2006; Dupuy and Simon, 2007).

The outline of the paper is as follows. In the section ‘Primary studies of progostic markers’, we consider primary studies of prognostic markers and consider their design, analysis, and reporting. In the section ‘Systematic reviews and meta-analysis’, we discuss systematic reviews of such studies and explain why meta-analyses that use individual patient data (IPD) are important. In the section ‘Towards multi-centre and collaborative research’, we consider why prospectively planned pooled analyses and the initiation of tumour banks may best facilitate evidence-based prognostic marker results. Finally, in the Discussion section, we summarise the key messages for future research.

## Primary studies of prognostic markers

### Study design

In the initiation of prognostic marker studies, as Altman and Lyman (1998) suggest, every effort must be made to limit potential biases and emulate the design standards expected of a protocol-driven randomised trial. Researchers should begin with a clear and well-defined research question so that their study can be designed and targeted appropriately. Unfortunately, it seems that many prognostic studies are not motivated by prior questions or by hypotheses, and few are protocol-driven – ‘investigators may tend to conduct opportunistic studies on the basis of specimen availability rather than on thoughtful design’ (Kyzas *et al*, 2007b). For example, often no justification is made for the sample size used, or whether the markers, sub-populations, and outcomes assessed were determined before beginning the study. It can be speculated that the main motivation for a quick analysis of some pre-existing data is another publication rather than scientific importance. It is, however, good practice for all research to have a study protocol outlining the aims and detailing the methods that will be used.

**Figure 1:**
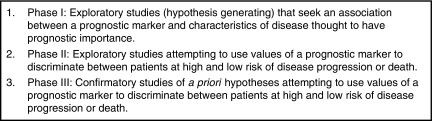
Types of prognostic marker studies, modified from Altman and Lyman (1998).

Prognostic marker studies have several possible aims, including the desire to understand more about the biology of a disease process. However, the vast majority of studies aim to investigate the association between one or more markers with an outcome (or multiple outcomes) of interest. In cancer, these outcomes are usually death, recurrence of disease, or both. For such studies, three different study phases have been proposed (Figure 1), which are similar in spirit to those proposed for biomarker development (Pepe *et al*, 2001; Ransohoff, 2007), and prognostic factor studies in general (Hayden *et al*, 2008). They begin with exploratory studies to identify promising prognostic markers and move towards larger, preferably prospective, confirmatory studies. Phase I studies are early exploratory analyses to generate hypotheses and to identify potential markers for further investigation. Phase II studies continue the exploratory investigation and assess the relationship between marker and prognosis. Phase III studies are large, confirmatory studies that state prior hypotheses, potentially based on earlier phase II results. These studies should certainly be protocol-driven and must be seen as the highest level of evidence that can arise from an individual prognostic study.

It is important for researchers to consider *a priori* which of these study types fit their research objective. There is nothing wrong with exploratory and hypothesis generating phase I or II studies. Furthermore, additional exploratory analyses of phase III studies are encouraged, as all such exploratory works help to identify potentially important markers and to validate measurement techniques. However, it is important that this type of research is clearly labelled and interpreted as such. After the publication of several small phase I or II studies, it seems useless, or even harmful, to proceed with further such studies for a particular marker of interest (Schmitz-Dräger *et al*, 2000). Research efforts should rather concentrate on larger collaborative phase III projects. The availability of a study register would help researchers to decide about starting further smaller studies or whether the time has come for a collaborative phase III study. Study registers would also prevent duplication of research effort, and allow transparency of *all* the markers that have been investigated, not just those found to be potentially important. Study registers are an integral part of randomised trials (Horton and Smith, 1999), thanks to many years of petitioning (Simes, 1986), and this sets the standard for transparent prognostic marker studies to follow. We note that such registers may be easier to achieve for phase III prognostic studies, which are pre-planned and protocol-driven by nature. On the contrary, a concern is that, owing to their opportunistic nature, many phase I or II studies would only be registered if they identify a significant finding, resulting in a biased set of registered phase I and II studies, akin to the problem of publication bias. Registration before the start of a study would be ideal, but we are aware that it will be difficult to achieve such a situation with phase I or II studies.

**Figure 2:**
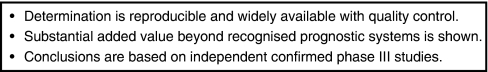
Requirements of a prognostic marker for acceptance in clinical practice, modified from Simon and Altman (1994).

**Figure 3:**
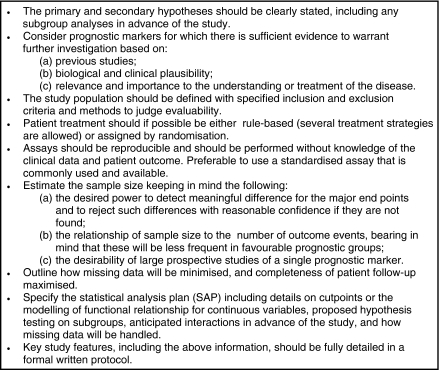
Factors to consider in the design of a high-quality phase III study, modified from Altman and Lyman (1998).

Unfortunately, the current literature indicates that phase I and II studies are by far the most common, with the higher-quality phase III studies unfortunately quite rare. This is disappointing, as results from phase III studies are more reliable and should underpin which markers are implemented in practice. Simon and Altman (1994) presented requirements of a prognostic marker for acceptance in clinical practice (Figure 2), and these include confirmation from phase III studies that the marker has independent prognostic ability beyond other markers used in practice (Kattan, 2003). Factors to consider in the design of phase III studies are shown in Figure 3. An ideal approach would involve following a well-defined cohort of patients from the same stage of their disease. Such a sample is often an ‘inception’ cohort of patients at the time of diagnosis of cancer. The use of study protocols, pre-specified study objectives, and prior hypotheses are essential. Detailed consideration should be given to the sample selection, inclusion and exclusion criteria, treatment (if possible randomised or standardised), the use of standardised assays, and statistical analysis (see later). For phase III studies, sample size should be large enough to sustain meaningful analysis, preferably with hundreds of events, and to ensure that the marker of interest can be assessed alongside existing markers of prognostic importance. Kyzas *et al* (2007b) highlight that power calculations for the required sample size are rarely presented in published prognostic marker studies. Procedures are also needed to ensure missing data are minimised and completeness of follow-up is maximised.

Although protocol-driven prospective studies are the ideal (Van Meerbeeck, 1994; Altman and Lyman, 1998), unfortunately the large majority of prognostic studies in cancer are retrospective and, it seems, not protocol-driven. A review of 331 prognostic marker studies identified only 20% that were prospective (Kyzas *et al*, 2007b), in the sense that the data were collected after the research question was posed. The big advantage of retrospective studies is the availability of a cohort with a long enough follow-up to assess a substantial number of outcome events (deaths or recurrences). Retrospective studies, however, have several serious disadvantages. Foremost among these are problems associated with the lack of a fully specified design for the study: unclear inclusion criteria, unknown completeness of the cohort, lack of standardisation of diagnostic and therapeutic procedures, incomplete baseline data, and unclear completeness of follow-up. A move towards prospective prognostic studies is thus encouraged, especially within phase III research. This may require collaboration among multiple research groups, so to achieve larger sample sizes and consistency across groups in important factors (e.g., measurement techniques, cutpoints used). Such studies can best be integrated in large therapeutic trials, but a protocol-based pre-planned pooled analysis of retrospective data from several centres is also possible (see the section ‘Towards multi-centre and collaborative research’).

### Statistical analysis

For therapeutic studies, statistical principles and methods are well developed and generally accepted. In contrast, no such consensus exists for the evaluation of prognostic markers. Holländer and Sauerbrei (2006) showed that the choice of statistical method has a strong influence on the results and, therefore, on the interpretation of prognostic marker studies. Focusing on prognostic markers in oncology, they discuss issues of statistical model building in the framework of regression models and of classification and regression trees. Other approaches, for example, artificial neural networks, are sometimes used for the analysis of marker studies but have several drawbacks (for more detailed discussion on this topic see Schwarzer *et al*, 2000; Schumacher *et al*, 2006). Parametric survival models are also possible, but are rarely used for the identification of prognostic markers.

For the analysis of a prognostic marker study, we consider multivariable regression models as the method of choice, such as the Cox model, which is often suitable for survival-type data that arises from prognostic studies. This assessment is based on both personal experiences and the results of simulation studies considering a restricted range of model-building issues (Sauerbrei, 1999; Royston and Sauerbrei, 2008). Multivariate analyses are easy to perform and provide prognostic marker results adjusted for other markers; this is imperative to assess the added value of a new marker over existing markers (Kattan, 2003), which should be the standard practice but is currently the exception in the prognostic marker literature (Kyzas *et al*, 2005).

Within regression models, a central issue is whether to use the full model incorporating all available variables or a reduced model determined by variable selection methods. Although it is well known that variable selection has several difficulties (Sauerbrei, 1999; Harrell, 2001), these strategies are often required to derive a sensible, interpretable, and parsimonious model (Sauerbrei, 1999; Royston and Sauerbrei, 2008). A more crucial issue is the way in which continuous markers are analysed. We believe it is central to make maximal use of the data and determine a sensible functional form (Royston *et al*, 2006; Sauerbrei *et al*, 2007; Royston and Sauerbrei, 2008). Researchers may retain a variable as a continuous measurement but they often assume a linear relation to outcome, which is unwise. More often they choose to avoid such issues by dichotomising, often at the sample median. This popular ‘step function’ approach contradicts biological thinking and has major methodological deficiencies, not least the reduced statistical power (Royston *et al*, 2006). Using the so-called ‘optimal cutpoint’ approach is the worst form of the step function approach, as it introduces considerable bias (Altman *et al*, 1994; Royston *et al*, 2006). Use of more than one cutpoint is better than having just one, but we believe that full use of the available data mandates a careful analysis that uses the actual continuous marker values (Holländer and Sauerbrei, 2006; Royston and Sauerbrei, 2008). Recommendations towards model building by the selection of variables and functional forms for continuous markers are available (Sauerbrei *et al*, 2007).

An important statistical topic that is often neglected in prognostic marker studies is model validation with external data (Holländer and Sauerbrei, 2006). Schumacher *et al* (2006) state that: ‘A multivariate approach is absolutely essential. Thoughtful application of model building techniques should help to obtain models that are as simple and parsimonious as possible and to avoid serious overfitting in order to achieve generalisability for future patients. Thus, validation in an independent study is a further essential step’. Unfortunately, most markers or combination of markers used as the basis for classification schemes never undergo external validation in a new data, and those that do rarely maintain their prognostic ability (Sauerbrei *et al*, 1997); consequently, many so-called prognostic markers are often not accepted for general use (Wyatt and Altman, 1995; Boracchi and Biganzoli, 2003). Altman and Royston (2000) propose that statistical and clinical validation of prognostic models is required, and they examine some general approaches to do this. One way of validating whether a marker truly is prognostic is to look at the results across multiple studies (meta-analysis), but this may be difficult without IPD (see section ‘The benefit of IPD’) (Tudur-Smith *et al*, 2005). Bennett (2003) provides a helpful review of analytic methods for analysing time-to-event data in single studies and meta-analysis, considering non-standard issues such as non-proportional hazards and heterogeneity.

Kattan (2003), and also Katz and Kattan (2005), highlights that the added value of new prognostic markers should be assessed not only by multivariate analyses but also through their ability to improve predictive accuracy. Two new measures applicable to models for binary outcome are proposed by Pencina *et al* (2008). Pepe *et al* (2004) also state that: ‘Markers proposed for classifying or predicting risk in individual subjects must be held to a much higher standard than merely being associated with outcome.’ Vickers and Elkin (2006) provide a novel method for evaluating prediction models, whereas Henderson and Keiding (2005) argue that although prediction models can be useful at the group or the population level, they are usually of no real use for individual patients because of the large uncertainty in human survival.

**Figure 4:**
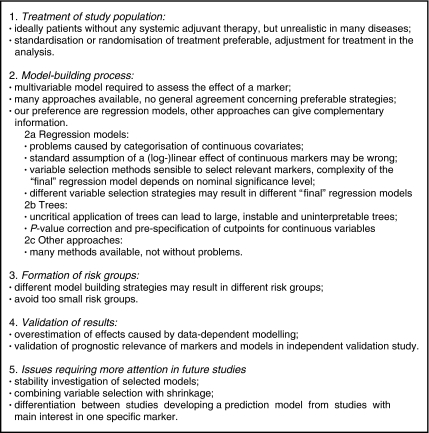
Summary of important issues for the analysis of single prognostic marker studies (Holländer and Sauerbrei, 2006).

In Figure 4, we reproduce the important issues for the analysis of a prognostic marker study from Holländer and Sauerbrei (2006). As for all high-quality, protocol-driven research, we recommend that the statistical analysis plan of a prognostic marker study should be specified in the study protocol and include details of how the independent prognostic importance of the marker will be ascertained in relation to existing markers of clinical importance. Furthermore, such pre-specified analyses should be complemented by sensitivity analyses of central assumptions and by additional model building using suitable strategies. These latter results are explanatory, which should be clarified in the study report.

### Study reporting

In addition to improving the design and analysis of primary studies, there is also a pressing need to improve their reporting standards, as serious deficiencies have been exposed (Altman *et al*, 1995; Riley *et al*, 2003; Burton and Altman, 2004; Kyzas *et al*, 2005, 2007a). Published studies currently lack sufficient information to allow a full appreciation of the methodological quality of the study, the methods, and analyses undertaken, or the applicability of the study results for practice. For instance, the following rudimentary factors are often not reported: the number of patients in each marker group; the number of events in each group; the method of measuring each marker; the cutpoint used to dichotomise a continuous marker into ‘high’ and ‘low’ levels; a measure of effect, such as a hazard ratio, and its confidence interval; marker results adjusted for other clinically useful markers; and which analyses, outcome, and markers were primary (pre-defined) objectives and which were exploratory secondary assessments (Riley *et al*, 2003). Kyzas *et al* (2007b) reviewed 331 cancer prognostic studies from 20 meta-analyses, and concluded that the reporting quality of study design and assay information was often suboptimal. Such gaps in reporting may explain why most systematic reviews of prognostic markers do not include an adequate quality appraisal of the studies identified (Hayden *et al*, 2006).

There is also evidence that prognostic results are often subject to selective reporting, meaning that some of the markers, outcomes, and analyses considered are not reported upon publication (Kyzas *et al*, 2005). This issue is associated with the common threat of publication bias (Sterne *et al*, 2001; Rothstein *et al*, 2005), where studies that do not identify statistically or clinically significant results are not published. Such publication bias is well recognised for randomised trials, even for Food and Drug Administration (FDA)-registered studies (Turner *et al*, 2008), and it seems certain that this bias also affects prognostic studies to a great extent. The biggest example so far was shown by Kyzas *et al* (2007a), who found that <1.5% of 1915 articles on cancer prognostic markers were fully ‘negative,’ in that they did not present statistically significant prognostic results and did not, for example, elaborate on nonsignificant trends. Selective publication is likely to lead to larger effects that are seen in smaller studies (Sterne *et al*, 2001; Rothstein *et al*, 2005). For example, in a systematic review of studies of Bcl2 in non-small-cell lung cancer, almost all the smaller studies showed a statistically significant relationship between Bcl2 and the risk of dying, whereas the three large studies were all nonsignificant and showed a much smaller effect (Martin *et al*, 2003). Simon (2001) commented that the prognostic literature ‘is probably cluttered with false-positive studies that would not have been submitted or published if the results had come out differently’. Such publication bias would thus lead to the literature being biased towards over-estimating the prognostic importance of markers.

**Figure 5:**
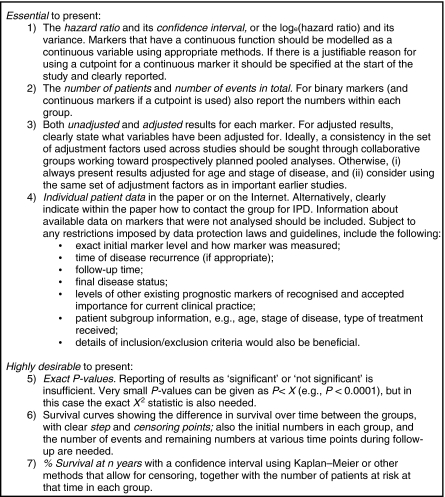
Guidelines for reporting the results of a prognostic marker study (Riley *et al*, 2003).

Rifai *et al* (2008) believe that it is time to take action against reporting biases in prognostic studies. To encourage clear and transparent reporting of prognostic marker studies, some recent articles have provided reporting guidelines. The REMARK guidelines (McShane *et al*, 2005b) consider the whole study process and suggest the key information that needs reporting, from the pre-defined hypotheses and patients included, to the statistical analysis methods used and the results identified, and to the study limitations and implications for clinical practice. Journal editors are encouraged to enforce adherence to these guidelines before accepting a prognostic marker article. In addition, Burton and Altman (2004) consider how to report prognostic studies when there are missing covariate data, whereas Riley *et al* (2003) suggest how effect estimates and summary results should be reported (Figure 5), with a recommendation to also provide IPD to facilitate meta-analysis.

## Systematic reviews and meta-analysis

### Current difficulties for evidence synthesis

An evidence-based approach to prognostic markers is clearly needed. It is usually difficult to ascertain the benefit of a marker from a single published study, which may be overoptimistic owing to small sample size and selective reporting, and a clear view is only likely to emerge from looking across multiple studies. However, clinicians do not have the time to review such a plethora of marker studies, and ideally need the most suitable prognostic markers to be identified for them, as exemplified by the American Society of Clinical Oncology in breast cancer (Harris *et al*, 2007). To aid this process, a systematic review is commonly used, which is an approach for identifying, evaluating and summarising an evidence-base. The term ‘systematic’ comes from the fact that the review process is performed using systematic and explicit methods, so that the review should be transparent and reproducible. If appropriate, a meta-analysis can be carried out at the end of a systematic review, which is a statistical approach that suitably combines the quantitative evidence from all the available studies (or from the subset of better-quality studies) to produce overall results for practice (Sutton *et al*, 2000).

Traditionally, meta-analysis methods use data extracted from published reports, but even for a summary assessment of a treatment effect from randomised trials, this approach has severe limitations (Piedbois and Buyse, 2004). Indeed, systematic reviews and meta-analyses of published prognostic studies generally highlight a confusing picture and are usually limited in their conclusions. As individual studies of prognostic markers are often poorly designed and poorly reported, the available evidence for synthesis is seriously limited. Also, multiple studies of a particular marker typically vary in important ways, such as the assays used, inclusion criteria for patients, types of treatment, cutpoint level, clinical outcomes, and statistical analysis. This methodological variation introduces heterogeneity between studies, and compounds the problem of summarising a marker's prognostic importance and identifying how to implement it in practice. For example, Parker *et al* (2001) performed a systematic review in prostate cancer to establish whether age is a prognostic marker, but the incomplete and heterogeneous nature of the reports prohibited any quantitative overview. Other issues that commonly hinder meta-analysis are the use of assays that are non-standardised or lacking in reproducibility; inappropriate or misleading statistical analyses; and optimal cutpoints, publication bias, and within-study selective reporting, all of which mean that the available published results are unlikely to represent a true picture. An assessment of the published results for marker MYCN in neuroblastoma strongly indicated that publication bias was present (Riley *et al*, 2004b), and the meta-analysis result obtained was likely to be an overestimate of the true prognostic effect for this marker. Furthermore, a recent review of the tumour suppressor protein, TP53, in head and neck cancer provides compelling empirical evidence that selective reporting biases are a major impediment to a meaningful meta-analysis (Kyzas *et al*, 2005). These biases have serious implications not only for meta-analysis but also for interpretation of the cancer prognostic literature as a whole (McShane *et al*, 2005a).

It is particularly important for systematic reviews of prognostic marker studies to appraise the methodological quality of the studies identified (Hayden *et al*, 2006). Often the main benefit of a systematic review of published prognostic studies is to expose the problems within primary research, and thus highlight the appropriate direction of future research. For example, Schmitz-Dräger *et al* (2000) reviewed 43 trials that considered p53 immunohistochemistry as a prognostic marker in bladder cancer, and concluded that: ‘From this analysis it becomes evident that further retrospective investigations will not contribute to the solution of the problem and thus are obsolete. There is an obvious need for standardisation of the assay procedure and the assessment of the specimens as well as for the initiation of a prospective multi-centre trial to provide definite answers.’ Individual prognostic studies are usually carried out independently and not in the context of facilitating a systematic review or a meta-analysis, either during the study process or when writing for publication. New studies should rather build specifically on the results of previous studies, forming a collective drive towards answering questions of real clinical importance. In particular, when a study assesses the potential of a new prognostic marker, it should do so in relation to those other markers identified earlier as important and currently used in clinical practice. For instance, the stage and grade of disease are used as prognostic indicators in most tumours, and therefore results for new markers need to be adjusted for these variables. However, in general, such adjusted results are not always reported and, even when they are, great inconsistency exists across studies regarding which factors are used for adjustment (Riley *et al*, 2003). This and other quality issues regarding the analysis of primary studies can be circumvented if IPD are available rather than only the published results, as now considered.

### The benefit of IPD

**Figure 6:**
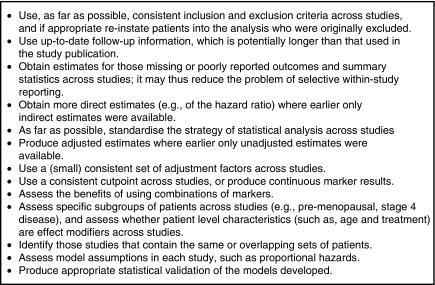
Summary of the potential benefits of having individual patient data (IPD) for a meta-analysis of prognostic marker studies.

The availability of IPD from primary prognostic studies offers many advantages for meta-analysis (Figure 6). For example, IPD allows the adequate checking of the data and modelling assumptions; unpublished results and outcomes to be obtained; an extended follow-up of patients; standardised analysis of the data with proper and consistent handling of continuous variables (Holländer and Sauerbrei, 2006; Royston *et al*, 2006); and suitable validation of the models developed (Altman and Royston, 2000; Holländer and Sauerbrei, 2006). It may also increase the opportunity to evaluate combinations of markers, which may produce more specific and accurate prognostic assessments than the individual markers themselves. We thus strongly recommend the IPD meta-analysis approach, but recognise that IPD may not solve all the problems (Stewart and Tierney, 2002), such as poorly designed primary studies. It may also be costly and time-consuming to obtain, or only available from a proportion of studies (Riley *et al*, 2007a, 2008).

The feasibility of obtaining IPD from prognostic studies has recently been considered by Altman *et al* (2006), and more generally for survival studies by Ioannidis *et al* (2002). Altman *et al* concluded that the IPD approach is possible, although it can be a ‘long, expensive, and rather laborious process’. To reduce the time and cost required, one option is to seek IPD from only a ‘well-defined list’ of studies, as successfully done by Look *et al* (2002) in breast cancer, although this approach may be criticised for potentially subjective decisions regarding what constitutes a ‘well-defined list’ and a ‘large’ or ‘high-quality’ study.

## Towards multi-centre and collaborative research

### Prospectively planned pooled analyses

It is clear that collaboration across multiple disciplines and multiple centres is required to achieve the necessary progress outlined in the sections ‘Primary studies of progostic markers’ and ‘Systematic reviews and meta-analysis’. However, such a collaborative drive naturally points away from the retrospective pooling of data, and points toward prospectively planned pooled analyses and prospective multi-centre studies. Indeed, McShane *et al* (2005a) state that ‘More importantly, the necessity of large, definitive prospective studies or prospectively planned meta-analyses for tumour marker research must be recognised.’ Research groups working together and communicating from the outset of their studies – as protocols and clinical objectives are being formulated – are the best way to achieve consistency in, for example, study design, markers assessed, method of measurement, treatments considered, outcomes of importance, and statistical analysis. A pooled analysis could then be planned prospectively to answer pre-specified clinical questions, with investigators committing in advance to the availability of their IPD at the end of their study.

Prospective multi-centre studies are the ideal, but may be time-consuming and costly. For prognostic markers measurable on stored material, a helpful compromise could be a protocol-based pre-planned pooled analysis of retrospective data from several centres. Such a project would start with a detailed protocol specifying inclusion and exclusion criteria for patients and treatments allowed. The availability of complete data for some ‘basic’ variables and a minimum amount of follow-up could be inclusion criteria. Compared with the effect of markers, the effect of most treatments is often relatively small. Therefore, the variation of treatment across several centres will not matter too much. The situation is more complicated if the treatment effect depends strongly on the marker (treatment–covariate interaction). However, that is rare. Such a situation requires a more thoughtful analysis, for example, a stratified analysis or separate subgroup investigations. Ideally, in all centres, the same method of the marker measurement has to be used, otherwise methods to transfer data to one scale are required, and other technical variations across studies are restricted as far as possible. Combining the estimated effects from the individual centres (studies) using meta-analysis methodology or by stratifying the analysis by centre (or treatment) gives the flavour of a pre-planned meta-analysis, a design used for some time in epidemiology (Blettner *et al*, 1999; Boffetta *et al*, 2004; Cardis *et al*, 2005). A combined analysis of data from several centres (studies), which is based on a detailed protocol before data collection is started, is an important design for future prognostic marker research. The required sample size can be reached by adding more centres, and the time frame for such a study can be ‘relatively’ short as data from patients with long-term follow-up can be used. As thousands of patients are required for a reliable assessment of the importance of a marker, it is necessary that large centres be willing to cooperate in such a project. Such an approach has many scientific advantages to a situation where large (and small) centres conduct studies on their own with hundreds of patients, but with variations in inclusion criteria, such as measurement techniques, analysis strategies, and so on. As obvious from the past, such unstructured type of research will not lead to scientific answers urgently needed. McShane *et al* (2005a) suggest ‘cultural changes will be required’, and the necessity of this type of collaboration has been recognised before by clinical trialists and by the epidemiologic community.

### Tissue banks

Another promising possibility is the use of samples from a tissue bank, especially one that is established to facilitate prognostic research (Burke and Henson, 1998; Schilsky *et al*, 2002). Tumour samples and corresponding data may come from patients treated within a prospective (randomised) trial, or they may come from those not taking part in a trial. In the earlier case, storing may be connected with a more specific project for a specified population; in the latter case, patients may belong to a very heterogeneous population with regard to characteristics, treatment, and follow-up. Studies using the resource of a tissue bank would benefit from standardised collection and storage of samples, as well as from good quality baseline and follow-up data. If new assays need to be performed, they can be carried out using standardised laboratory methods. After the first few years, studies using such samples would have access to adequate clinical follow-up, and so could be carried out quickly but without the disadvantages associated with retrospective studies.

Hayes *et al* (2008) concur that the exciting potential of prognostic markers highlights the ‘importance of prospective collection, processing, and storage of biospecimens’ to help identify markers that facilitate individualised treatment strategies. A highly commendable example of such an initiative is in the bladder cancer field, where Goebell *et al* (2004) are establishing a multi-institutional bladder cancer database and a virtual tumour bank as a resource for participating institutions to evaluate the biological and prognostic significance of potential markers. More generally, the Confederation of Cancer Biobanks (CCB) are developing biobank resources for cancer research and promoting consistency in how tissue samples are prepared and preserved, thus to facilitate research investigations involving a larger number of patient samples. Arguments against tissue banks usually relate to confidentiality or to medico-legal concerns, but increasingly tissue banks are accompanied by strong legal and ethical requirements, and require patient consent. A discussion of the current ethical issues involving tissue banks is reported by Ravid (2008).

Future single and multi-centre prospective prognostic marker studies can also facilitate the initiation of tissue banks by providing their patient tissue samples and archiving their IPD of each patient's baseline covariates, clinical follow-up, and eventual outcomes. This would allow further investigations in the light of new information after the studies ended; for example, if subsequently a new marker is discovered, then the tissue samples could be assessed for this marker and comparison made to the existing markers. Data from such a marker can be most relevant to the investigations of treatment–covariate interactions in a (single) large randomised trial, but it can also be used in prospectively planned pooled analyses as noted above. For a recent example investigating several markers for an interaction with treatment in a lung cancer trial, see Olaussen *et al* (2006) and Filipits *et al* (2007a, 2007b).

## Discussion

Any insight into the future health of an individual patient is advantageous, and so prognostic markers can potentially play a vital part in clinical decision-making (Windeler, 2000). It is imperative, therefore, that researchers produce reliable and informative evidence regarding the prognostic markers available for practice. However, for whatever reason, it is clear that prognostic marker studies have been neglected in the drive to improve medical research. The responsibility for this lies with all those involved in prognostic research, from those identifying and measuring markers in the laboratory, to those designing and implementing primary studies, to those analysing and reporting results, and to those reviewing and publishing studies. We must now work together across multiple disciplines, and move towards transparent, high-quality, clinically relevant research relating to prognostic markers. Higher-quality prognostic marker research is desperately needed and is attainable. To achieve such progress, collaborative and multi-disciplinary teams should be an essential part of future prognostic studies, including clinicians, biologists, and statisticians, among others. Research on treatment strategies has successfully involved international and multi-disciplinary collaborations for many years, and this is undoubtedly the way forward for prognostic marker research. Indeed, the best chance of answering important questions about prognosis is likely to come from ‘a systematic, sustained programme of epidemiologic research, coordinated among cognate research groups’ (Hemingway, 2006). To this end, we encourage researchers to join the newly registered Prognosis Methods Group within the Cochrane Collaboration (Riley *et al*, 2007b).

**Figure 7:**
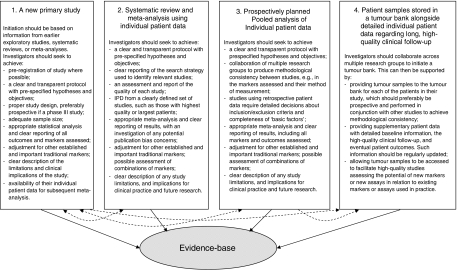
Pathways to high-quality evidence regarding the prognostic ability of a marker, following publication of initial hypothesis generating studies.

In this paper, we have encouraged progress towards higher-quality prognostic studies by explaining where current deficiencies lie, and by discussing some existing guidelines regarding the design, clinical relevance, analysis, and reporting of such studies. Hemingway (2007) recommends that: ‘We need not only to develop quality standards of primary, secondary, and tertiary prognosis research but also to secure their implementation’. Cultural changes thus need to be embedded in the research community, with continued dissemination of best practice made within and across research groups, and at national and international meetings. A pivotal role in ensuring good practice is held by the editors of and reviewers for clinical journals, who can enforce certain standards. For example, some journals require the prospective registration of randomised controlled trials (De Angelis *et al*, 2004), and this is an option worth exploring further for prognostic studies. Researchers also need to look beyond single prognostic studies and consider the bigger picture: the clinical utility of a marker will only be established through multiple high-quality studies conducted over a period of time. Prospective phase III studies, prospectively planned meta-analyses of prospective or retrospective data, the availability of IPD, and the initiation of tumour banks all support the evolution of prognostic marker evidence. In Figure 7 we summarise this process and indicate the different stages in which researchers can make a significant contribution to a marker's evidence-base. Research groups should strive for such opportunities, and make a long-standing commitment towards high-quality and collaborative studies in the future. Only then will we achieve the evidence-based use of markers in practice and ensure the most appropriate patient care, which should be the aim of all of us.
